# Reduction Impairs the Antibacterial Activity but Benefits the LPS Neutralization Ability of Human Enteric Defensin 5

**DOI:** 10.1038/srep22875

**Published:** 2016-03-10

**Authors:** Cheng Wang, Mingqiang Shen, Naixin Zhang, Song Wang, Yang Xu, Shilei Chen, Fang Chen, Ke Yang, Ting He, Aiping Wang, Yongping Su, Tianmin Cheng, Jinghong Zhao, Junping Wang

**Affiliations:** 1 State Key Laboratory of Trauma, Burns and Combined Injury, Institute of Combined Injury of PLA, Chongqing Engineering Research Center for Nanomedicine, College of Preventive Medicine, Third Military Medical University, Chongqing, 400038, China; 2 Department of Nephrology, Xinqiao Hospital, Third Military Medical University, Chongqing, 400037, China

## Abstract

Oxidized human defensin 5 (HD5_OX_), a Paneth cell-secreted antibacterial peptide with three characteristic disulfide bonds, protects the host from invasion by morbigenous microbes in the small intestine. HD5_OX_ can be reduced by thioredoxin (Trx) *in vitro*, while the biochemical properties of the reduced linear peptide, HD5_RED_, remain unclear. Here, we first confirm that HD5_RED_ does exist *in vivo*. Furthermore, we reveal that the recruitment of HD5_RED_ to the outer membrane of Gram-negative bacteria and to the anionic lipid A is lower than that of HD5_OX_, and HD5_RED_ is less efficient in penetrating bacterial outer and inner membranes and inducing membrane depolarization, which confers an attenuated antibacterial activity to HD5_RED_. However, due to its higher structural flexibility, the binding of HD5_RED_ to bacterial lipopolysaccharide (LPS) is markedly stronger than that of HD5_OX_. Consequently, HD5_RED_ is more effective in suppressing the production of the pro-inflammatory cytokine TNF-α in LPS-stimulated macrophages by blocking the interaction between LPS and LPS-binding protein, thus suggesting that HD5_RED_ might act as a scavenger to neutralize LPS in the gut. This study provides insights into the antibacterial and immunoregulatory effects of HD5_RED_ and expands the known repertoire of the enteric defensins.

Defensins are small (2–5 kDa), Cys-rich, endogenously produced, amphiphilic peptides that serve a fundamental role in the first line of host immune defence against invading microbes^1^. Based on their sequence homology and disulfide pairings, human defensins are classified into two major subfamilies, α and β^2^,^3^, which are primarily discovered in neutrophils and epithelia, respectively^4^,^5^.

Oxidized human defensin 5 (HD5_OX_), a 32-residue α defensin, is stored in the secretory granules of Paneth cells in the small intestine as a propeptide (E20-R94) and is processed by anionic and/or meso isoforms of trypsin during secretion or shortly thereafter at a cleavage site between R62 and A63^6^. Because HD5_OX_ exhibits the widest spectrum of antibacterial activity among the α defensins^7^, it is a promising candidate that may be used to target pathogens that are clinically resistant to traditional antibiotics^8^. We have previously prepared bioactive HD5_OX_ using *Pichia pastoris* and designed a more potent antibiotic peptide based on its active region^9^,^10^. Recently, the thioredoxin (Trx) system was found to catalyze the reduction of intestinal defensins^11^,^12^. Notably, the antibacterial activity of human β defensin (HBD) 1, a ubiquitously expressed peptide in human epithelia, is enhanced upon disulfide reduction^13^. Based on the abundance of HD5_OX_ in the intestine^14^, the impact of disulfide reduction on its antibacterial activity warrants investigation.

Human defensins are also noted for their immunoregulatory properties. For instance, HBDs have been shown to recruit immature dendritic cells, memory T cells, macrophages, and monocytes via G_i_-protein-coupled receptors^15^,^16^, and human neutrophil peptides (HNPs) are capable of chemoattracting CD4^+^/CD45RA^+^ naive and CD8^+^ T cells^17^. Enteric HD5_OX_ can induce the production of IL-8 and IL-2 from intestinal epithelial cells and CD4^+^T cells, respectively^18^,^19^. Additionally, some defensins are able to suppress the pro-inflammatory effect of bacterial lipopolysaccharide (LPS). For example, HBD1 and HNP1 can block the interaction between LPS and low concentrations of LPS-binding protein (LBP), which is the initial step of LPS-TLR4 signalling, thus inhibiting the production of TNF-α from LPS-stimulated macrophages^20^. LPS is a potent elicitor of inflammation and sepsis^21^, yet it strongly induces the production of enteric defensins^22^. Because the defective expression of human defensin 5 (HD5) is related to diarrhoea^23^ and inflammatory bowel disease (IBD)^24^, two enteric disorders that are associated with an overreaction of host cells to LPS^25^,^26^,^27^, we speculated that HD5 may antagonize LPS *in vivo*.

In this study, we found that HD5_OX_ was less efficient in neutralizing LPS than HD5_RED_. Similar to other synthetic innate defence regulator peptides (IDRs)^28^, HD5_RED_ exhibited a structural flexibility that appears to be tailored to binding LPS and blocking the interaction between LPS and low concentrations of LBP. However, HD5_RED_ killed bacteria less efficiently than HD5_OX_. We investigated the underlying mechanism by measuring the recruitment of HD5_RED_ to bacteria and anionic lipid A, outer and inner membrane penetration, and changes in membrane potential. The results obtained indicate that HD5_OX_ reduction may benefit immunoregulation at the expense of bactericidal attenuation.

## Results and Discussion

### HD5_RED_ exists in the small intestine

**Figure 1:**
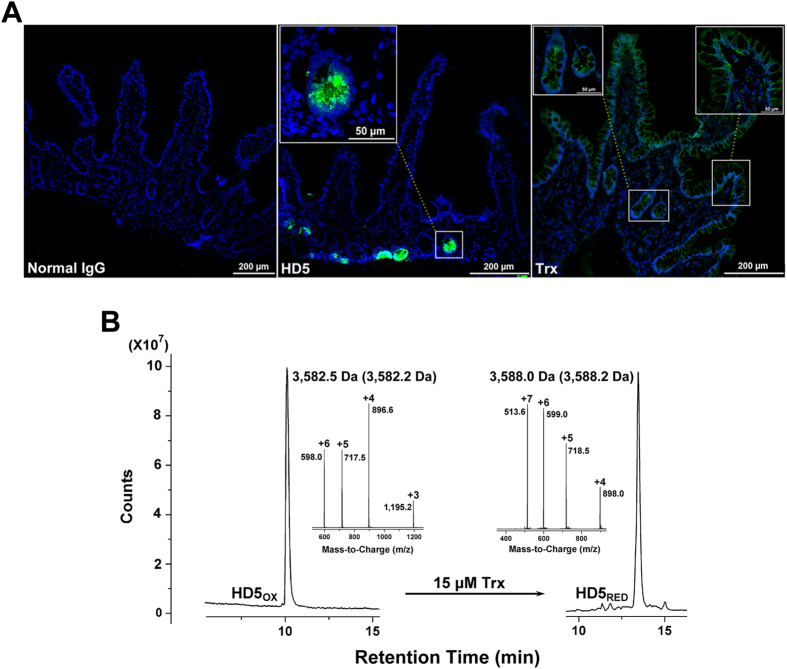
Distributions of HD5 and Trx in intestinal mucosa benefit the formation of HD5_RED_. (**A**) Immunofluorescence microscopy revealing the distributions of HD5 and Trx in human ileal mucosa. The target proteins were probed by a goat anti-rabbit Alexa Fluor 488 antibody. Nuclei were stained using DAPI (blue). The normal rabbit IgG was used as a negative control. Scale bar indicates 200 μm. The regions of interest are magnified in embedding graphs, in which the scale bar indicates 50 μm. (**B**) Trx catalyzed the reduction of HD5_OX_ *in vitro*. Values provided inside and outside the parentheses are theoretical and observed masses of HD5_OX_ and HD5_RED_, respectively. Theoretical mass was determined by ProtParam (http://web.expasy.org/protparam/).

We initially employed an immunofluorescence microscopy to observe the distributions of HD5 and thioredoxin (Trx), a multifunctional oxidoreductase that can catalyze the reduction of human defensins^11^,^12^,^13^, in the ileum. As shown in Fig. 1A, HD5 was located at the base of the intestinal crypts, and Trx was found in both the mucosal surface and crypts. The local distributions of HD5 and Trx provide a conducive condition for the formation of HD5_RED_. Because the storage of HD5 in human ileal mucosa is approximately 90–250 μg/cm^2^, we then used 30 μM (107.5 μg/mL) HD5_OX_ in a recently reported reduction condition to mimic the interaction between HD5_OX_ and Trx *in vitro*^12^, resulting in a product with increased hydrophobicity (Fig. 1B). The liquid chromatography-mass spectrometry (LC-MS) system confirmed the existence of HD5_RED_, as the molecular masses differed by approximately 6 Da (3,582.5 Da for HD5_OX_, 3,588.0 Da for HD5_RED_), corresponding to the reduction of three disulfide bonds. It is plausible that the reduction of Trx to the disulfide bonds of HD5_OX_ may contribute to the production of HD5_RED_ *in vivo*.

**Figure 2:**
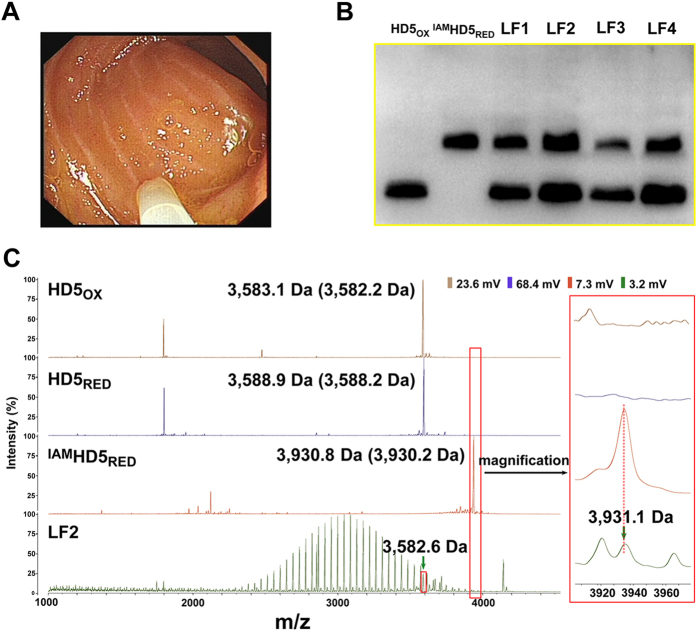
HD5_RED_ exists in human LF. (**A**) LF sample was obtained at the terminal ileum of healthy donor using an enteroscope equipped with a sterile catheter. (**B**) AU-PAGE immunoblotting. Four LF samples obtained from different donors were purified, lyophilized, and dissolved with 200 μL of the 5% acetic acid solution, which were named as LF1, LF2, LF3, and LF4, respectively. A total of 20 μL of each sample was resolved by 15% AU-PAGE. HD5_OX_ and ^IAM^HD5_RED_ (500 ng/lane) were employed as the positive controls. Shown is a cropped figure, in which the cropping lines are coloured yellow. The full-length blots are presented in Supplementary Fig. 1. (**C**) MALDI-TOF demonstrating the existence of HD5_RED_ in LF. Aliquot of 1 μL of LF2 was mixed with α-cyano-4-hydroxy cinnamic acid (CHCA, v/v, 1:1), co-crystallized, and analyzed with a MALDI-TOF. ^IAM^HD5_RED_ was prepared by incubating 20 μL of HD5_RED_ (1 mg/mL) and 5 μL of IAM (50 mM) in dark at room temperature for 2 h. Aliquots of 1 μL of ^IAM^HD5_RED_, 1 μL of HD5_OX_ (1 mg/mL), and 1 μL of HD5_RED_ (1 mg/mL) were employed as the positive controls. The mass spectrum around 3,931 Da is magnified in embedding graphs. Values provided inside and outside the parentheses are theoretical and observed masses, respectively.

To determine whether HD5_RED_ exists *in vivo*, we obtained the luminal fluid (LF) from the terminal ileum of four healthy donors underwent enteroscope (Fig. 2A). Once removed, the LF samples were alkylated by iodoacetamide (IAM) and analyzed by acetic acid urea-polyacrylamide gel electrophoresis (AU-PAGE) immunoblotting with a rabbit polyclonal anti-HD5 antibody. We observed that there were two bands in LF migrating in similar manners to HD5_OX_ and IAM-alkylated HD5_RED_ (^IAM^HD5_RED_), respectively (Fig. 2B, Supplementary Fig. 1), and that co-loading ^IAM^HD5_RED_ and LF resulted in a single but wider band in the region of ^IAM^HD5_RED_ (Supplementary Fig. 2). Furthermore, in the mass spectrum (1,000–4,500 Da) of LF obtained by the matrix-assisted laser desorption/ionization-time of flight mass spectrometry (MALDI-TOF), we observed a polyethylene glycol (PEG)-like signal around 3,000 Da^29^. It is highly possible that the laxative taken before enteroscope was attributable. Nevertheless, we identified two signals with molecular masses of 3,582.6 Da and 3,931.1 Da (Fig. 2C), which was similar to that of HD5_OX_ (3,583.1 Da) and ^IAM^HD5_RED_ (3,930.8 Da), respectively. Of note, the signal intensity of ^IAM^HD5_RED_ was much lower than that of HD5_OX_, which was different from the observation in AU-PAGE immunoblotting. This result might have occurred because the ionization efficiency of ^IAM^HD5_RED_ in LF was lower than that of HD5_OX_ in MALDI-TOF.

Previously, it was discovered that due to the cleavage of trypsin to propeptide, HD5 existed as unprocessed peptide in intestinal tissue and mature peptide in LF^6^. Our data expanded the result that mature HD5 existed as HD5_OX_ and HD5_RED_ in LF. The roles of defensin peptides in host immunity have been widely studied^7^,^30^,^31^, but little attention was paid to the reduced form of defensins. Along with the finding that HBD1_RED_ exists in human colon, ileum, and skin^13^, our study supported the existence of HD5_RED_ in the small intestine. As demonstrated previously^12^, the presence of zinc ions in the local area may protect HD5_RED_ from digestion by the enzymes in the enteric canal, although linear peptides are theoretically more susceptible to degradation by digestive enzymes.

### Reduction attenuates the antibacterial activity of HD5_OX_

**Figure 3:**
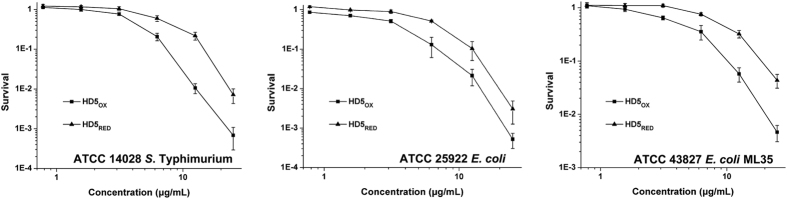
Reduction attenuates the antibacterial activity of HD5_OX_ against Gram-negative bacteria. Three Gram-negative strains, *Salmonella enterica* serovar Typhimurium (*S.* Typhimurium, ATCC 14028), *Escherichia coli* (*E. coli*, ATCC 25922), and *E. coli* ML35 (ATCC 43827), were exposed to HD5_OX_ and HD5_RED_, respectively. Peptides were prepared in sterile water, with the final concentrations of 0.78, 1.56, 3.13, 6.25, 12.5, and 25 μg/mL. The antibacterial activity is shown as the ratio of the number of surviving colonies after treatment with peptides to the number of surviving colonies after treatment with sterile water (HD5_OX_) or DTT solution (HD5_RED_, 0.016, 0.032, 0.063, 0.13, 0.25, and 0.5 mM DTT, respectively). The results are presented as the mean ± SD. Zero survival points are not plotted.

HD5_OX_ is an endogenous bactericidal agent. To investigate the effect of disulfide reduction on the antibacterial activity of HD5_OX_, we subsequently performed a virtual colony count assay in which three Gram-negative strains, *Salmonella enterica* serovar Typhimurium (*S.* Typhimurium, ATCC 14028), *Escherichia coli* (*E. coli*, ATCC 25922), and *E. coli* ML35 (ATCC 43827) were focused. Generally, HD5_OX_ exerted a more potent action against these bacteria than HD5_RED_, especially at peptide concentrations greater than 3.125 μg/mL (Fig. 3). The concentrations of HD5_RED_ needed to inactivate 90% of these bacteria were 19.5 ± 1.6, 12.9 ± 1.3, and 22.5 ± 2.3 μg/mL, which were 2.0-, 1.7-, and 1.9-fold higher than those of HD5_OX_, respectively (Table 1). The efficiency of HD5_RED_ against Gram-positive *Enterococcus faecalis* (*E. faecalis*) was less than HD5_OX_ as well (Supplementary Fig. 3). Notably, this finding differs from the results obtained for HBD1, in which the antibacterial activity is enhanced upon reduction^13^. This result might be due to the diverse structure-activity relationships observed between α and β defensins. For example, HBD3 can function well without disulfide bonds^32^, whereas loss of disulfide bonds attenuates the antibacterial property of HNP1^33^.

**Figure 4:**
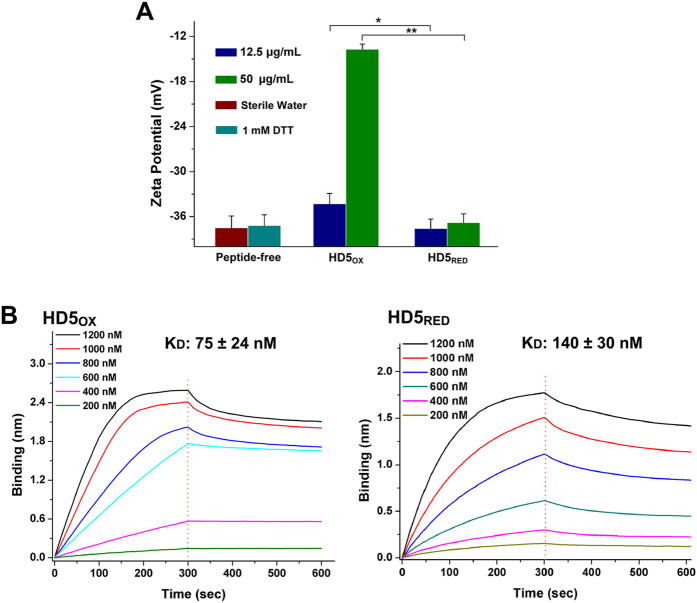
Reduction attenuates the recruitment of HD5_OX_ to bacterial membrane. (**A**) Zeta potential determination of *E. coli* ML35 cells exposed to HD5_OX_ and HD5_RED_. At 12.5 and 50 μg/mL, HD5_RED_ peptides are both less efficient in altering the bacterial surface charge than HD5_OX_. *p < 0.05; **p < 0.01. (**B**) BLI-based binding kinetics of HD5_OX_ and HD5_RED_ to Lipid A. The binding thicknesses of peptides at 200, 400, 600, 800, 1000, and 1200 nM were processed by subtracting the reference (bindings in 5 mM sodium phosphate buffer), respectively. Red dotted lines divide the kinetic curves into the association and disassociation portions. The K_D_ values are shown as the mean ± SD from three independent assays.

Existing data demonstrate that the antibacterial action of defensins is related to their recruitment to bacterial membranes and the subsequent membrane destruction^30^,^34^. To gain deeper insights into the observed loss of antibacterial activity, we measured the surface charge of *E. coli* ML35 cells treated with peptides. The zeta potential of cells in the peptide-free condition or in the presence of HD5_RED_ was approximately −37 mV, which was significantly less than that of cells treated with HD5_OX_ (Fig. 4A). Furthermore, we conducted a biolayer interferometry (BLI) to analyze the recruitment of HD5_RED_ to lipid A, which is considered a target for defensins on the outer membrane of Gram-negative bacteria^35^. Consistent with the idea that an integrated tertiary structure is needed for HD5_OX_ to efficiently bind and inhibit anthrax lethal factor (LF) and HIV gp-120^33^, we found that given a fixed concentration (1000 nM), fewer HD5_RED_ than HD5_OX_ molecules were recruited to the lipid A loaded on the amine-reactive second-generation (AR2G) biosensors in the presence of 5 mM sodium phosphate buffer (Fig. 4B). The equilibrium dissociation constant (K_D_) of HD5_RED_ binding to lipid A was 140 ± 30 nM (Supplementary Table. 1), which was 1.9-fold higher than that of HD5_OX_ (75 ± 24 nM), suggesting that the ability of HD5_OX_ to bind bacteria is impaired by disulfide reduction.

**Figure 5:**
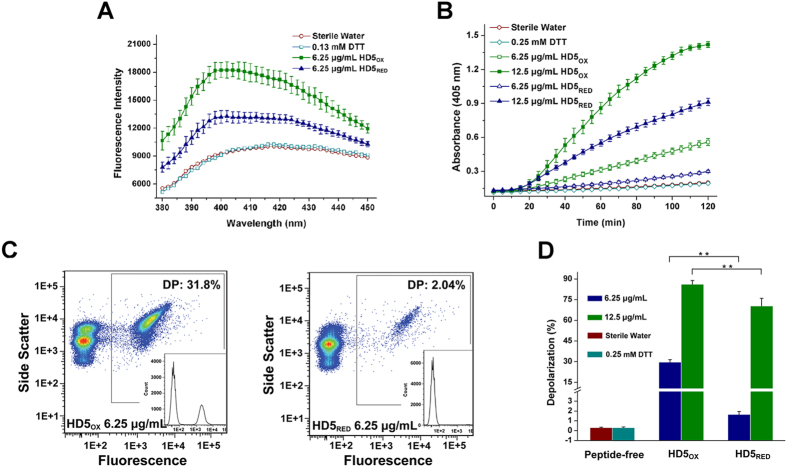
Reduction attenuates the interaction of HD5_OX_ with bacterial membrane. (**A**) Outer membrane penetration. *E. coli* ML35 cells were treated with the peptides (6.25 μg/mL), sterile water, and 0.13 mM DTT, respectively. The fluorescence intensity of NPN is displayed as the mean ± SD from three independent assays. To distinguish the effects of sterile water and DTT, the error bars are not shown. (**B**) Inner membrane permeabilization. *E. coli* ML35 cells were exposed to the peptides (6.25 and 12.5 μg/mL), sterile water, and 0.25 mM DTT, respectively. The results are shown as the mean ± SD. (**C**) Representative flow cytometry scatter plots of *E. coli* ML35 cells treated with 6.25 μg/mL of HD5_OX_ and HD5_RED_. (**D**) Histogram revealing the less efficiency of HD5_RED_ in inducing membrane depolarization relative to HD5_OX_. The mean depolarization values represent three independent experiments. The error bars indicate the SD. **p < 0.01.

To analyze the membrane destruction of bacteria exposed to peptides, we first performed a 1-N-phenylnaphthylamine (NPN) uptake assay, in which the *E. coli* ML35 strain and 6.25 μg/mL of HD5_OX_ and HD5_RED_ were used. NPN is a hydrophobic dye, and its fluorescence intensity is increased when it is incorporated into the outer membrane of disintegrated bacteria. As the fluorescence intensity of HD5_RED_-treated cells was lower than that of HD5_OX_-treated cells (Fig. 5A), it is evident that HD5_RED_ destroys the outer membrane less effectively than HD5_OX_. Because *E. coli* ML35 lacks lactose permease, this bacterium is often employed to determine the effect of antibiotic peptides on the bacterial inner membrane by measuring the production of 2-nitrophenol (ONP) generated from the hydrolyzation of cytoplasmic β-galactosidase to 2-nitrophenyl β-D-galactopyranoside (ONPG)^36^. We then performed an inner membrane permeabilization assay and found that, consistent with the findings of a study that native disulfide bonds are required for the efficient membrane penetration by HD5_OX_^37^, HD5_RED_ exerted an attenuated potency for disintegrating the bacterial inner membrane (Fig. 5B).

Previously, a study on the interaction of defensin peptides with *E. coli* ML35 showed that membrane destruction slightly lagged behind cell death at the early stage^38^, which may be due to the fact that the initial disruption of the membrane potential affects osmotic regulation and bacterial respiration^30^. It can be reasoned that in addition to measuring mechanical disruption, detecting the membrane potential change is also important for understanding the effect of defensins on the bacterial membrane. Therefore, we employed the bis-(1,3-dibutylbarbituric acid) trimethine oxonol [DiBAC4(3)], a potential-sensitive dye, and conducted a flow cytometry assay. The result showed that the ability of HD5_RED_ to induce membrane depolarization was significantly lower than that of HD5_OX_ (Fig. 5C,D), which indicates that HD5_RED_ cannot efficiently interact with the bacterial membrane.

**Figure 6:**
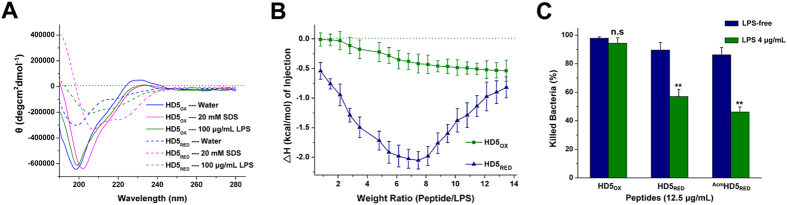
The improved structural flexibility allows the LPS-binding of HD5_RED_. (**A**) Circular dichroism spectra of HD5_OX_ and HD5_RED_ (100 μg/mL) in sterile water and in the solution containing 20 mM SDS or 100 μg/mL of LPS. (**B**) Heat release of the interaction between 1 mg/mL of the peptides and 10 μg/mL of LPS. Shown is the relationship between the enthalpy value (△H) and the weight ratio of peptides to LPS. The results are presented as the mean ± SD. (**C**) Impact of 4 μg/mL of LPS on the antibacterial activities of peptides (12.5 μg/mL) against *E. coli*. The antibacterial efficiency is presented as the bacterial lethal rate, which is calculated by dividing the number of killed colonies by the number of colonies remaining after treatment with sterile water or 0.25 mM DTT. Statistical significance was determined by the Student’s t-test. n.s, not significant; **p < 0.01.

Changes in function follow changes in structure. Circular dichroism (CD) spectroscopy revealed that HD5_RED_ is less effective in forming rigid β-sheet structures in aqueous, as demonstrated by an increased signal intensity at 200 nm, which is indicative of an increase in random coil structures (Fig. 6A). Moreover, in the presence of sodium dodecyl sulfate (SDS) micelles, a model used to mimic the prokaryotic membrane^39^, HD5_OX_ maintained the β-sheet structures, whereas HD5_RED_ transformed into a peptide containing α-helical structures, manifested by a strong positive peak at wavelengths between 190 and 193 nm and double minima at wavelengths of 205 and 220 nm. This result might have occurred because without the constraint of three disulfide bonds, the hydrophobic residues of HD5_RED_ were exposed to the solution and hydrophobically interacted with the lipid membrane, resulting in a conformational transformation. It can be supported by that due to the flexible N-terminus (Gly^1^ to Tyr^10^), HBD3 adopts a α-helical-containing structure in SDS micelles despite the β-sheet conformation^40^. Because a rigid structure is beneficial for the linear peptide in the destruction of Gram-negative bacteria^18^, we propose that the loss of antibacterial activity of HD5_RED_ is related to its structural flexibility.

### HD5_RED_ binds and neutralizes LPS

Upon examining the biochemical properties of synthetic IDRs, the structural flexibility was noted. For example, the bovine bactenecin linear derivative 1018 adopts various folds in different media, relative to the natural peptide, to adapt to different functions^28^. Additionally, the linear analogue of horse shoe crab *Tachypleus tridentatus* Tachyplesin-1 can transform its conformation in LPS micelles, which contributes to its LPS-binding and -neutralization abilities^41^. We therefore analyzed the secondary structures of HD5_OX_ and HD5_RED_ in the presence of 100 μg/mL of LPS and found that the spectrum of HD5_OX_ was slightly red-shifted (Fig. 6A). Comparatively, HD5_RED_ adopted a different conformation with two negative ellipticities at 217 nm and 206 nm, which is indicative of a β-sheet like structure^41^.

To determine the effect of the conformational transformation on LPS-binding, we performed an isothermal titration calorimetry (ITC) and discovered that similar to a recent study demonstrating that HD5_OX_ can bind LPS^42^, HD5_OX_ (1 mg/mL) exothermically interacted with 10 μg/mL of LPS (Fig. 6B). However, unlike the enthalpy value (△H), a parameter that reflects the contributions of van der Waals interaction force and hydrogen bond in intermolecular interactions, tends to decrease in titrations of representative binding signals (Supplementary Fig. 4), the △H progressively increased. It can be explained by that the binding response between HD5_OX_ and LPS is so weak that the heat release is counteracted by conformational alterations (heat absorption). For HD5_RED_, despite the greater conformational alterations, the heat release was higher than that of HD5_OX_. Moreover, as shown by a representative binding signal at weight ratios ≥8.0, the LPS-binding of HD5_RED_ seems to be much stronger than that of HD5_OX_. The results of an antibacterial assay supported that the efficiency of 12.5 μg/mL of HD5_OX_ was virtually unaffected by the presence of 4 μg/mL of LPS, whereas the activities of HD5_RED_ and ^Acm^HD5_RED_, an analogue of HD5_RED_ in which the cysteine sulfhydryls are protected by acetamidomethyl (Acm), were significantly impaired (Fig. 6C).

LPS is localized in the outer layer of Gram-negative bacteria and is released during bacterial growth and disintegration^43^. The active centre of LPS is lipid A, whose hydrophilic backbone and hydrophobic region are responsible for the specific binding to recognition molecules and the activation of cells, respectively^44^. By interacting with LPS-binding protein (LBP), lipid A is extracted from LPS micelles and transferred to membrane CD14^45^, which initiates the Toll-like receptor 4 (TLR4) signalling pathway and activates the transcription factor NF-κB, leading to the production of TNF-α, IL-6, and other inflammatory mediators^46^. This phenomenon is characteristic of host innate immune responses to bacterial pathogens. However, the uncontrolled production of these cytokines would likely contribute to morbidity as a result of endothelial damage, coagulopathy, and systemic inflammatory response syndrome (SIRS)^21^.

**Figure 7:**
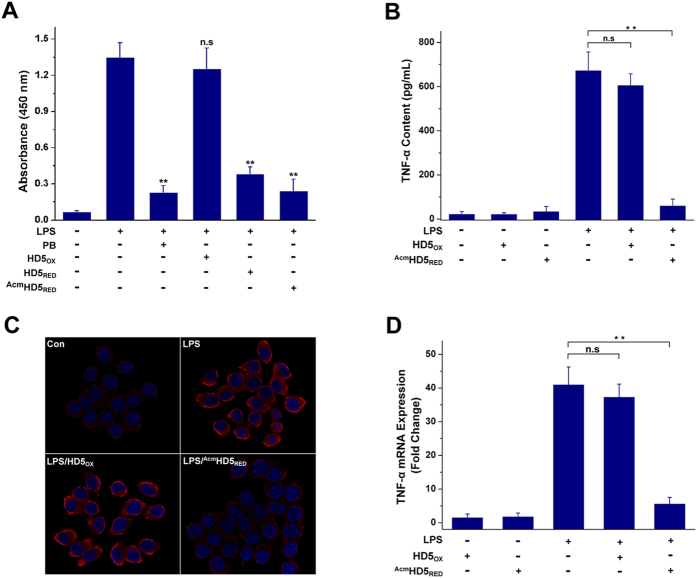
HD5_RED_ lowers the initiation of LPS-TLR4 signalling. (**A**) LBP block assay. Peptides were prepared in sterile water, with a final concentration of 50 μg/mL. Polymixin B (200 ng/mL) was used as a positive control. Statistical significance was determined by the Student−Newman−Keuls multiple comparisons test. n.s, not significant; **p < 0.01, relative to the value of the group solely treated with LPS. (**B**) ELISA was used to detect the TNF-α content in the supernatant of RAW264.7 cells. After adherence, cells were incubated with 5 ng/mL of LPS for 9 h in the absence or presence of 50 μg/mL of the peptides. The results are shown as the mean ± SD. n.s, not significant; **p < 0.01. (**C**) Immunofluorescence microscopy showing TNF-α in the cytoplasm of RAW264.7 cells. TNF-α was probed by a goat anti-mouse Alexa Fluor 555 antibody. The nuclei (blue) were stained using DAPI. (**D**) Quantitative RT-PCR illustrating that ^Acm^HD5_RED_ suppressed the production of TNF-α at the transcriptional level. n.s, not significant; **p < 0.01.

Because most antibiotics promote the release of LPS while killing bacteria^43^, much research has been devoted to the development of novel compounds that simultaneously inactivate bacteria and sequester LPS^47^. Antimicrobial peptides are promising candidates as some of them can suppress the initiation of TLR4-NF-κB signalling by binding LPS and blocking the interaction between LPS and low concentrations of LBP^20^,^48^. As HD5_RED_ was more potent than HD5_OX_ in LPS-binding, we further conducted a LBP block assay in which 200 ng/mL of polymixin B (PB) was employed as a positive control. The result showed that 50 μg/mL of HD5_OX_ had less effect on the interaction between LPS and LBP. Comparatively, PB, HD5_RED_, and ^Acm^HD5_RED_ significantly inhibited the LPS-LBP interaction (Fig. 7A). The dose-dependent LBP-blocking was also observed for HD5_RED_ and ^Acm^HD5_RED_ (Supplementary Fig. 5).

Because HD5_RED_ is prone to oxidation during the 9 h of *in vitro* incubation, we subsequently employed ^Acm^HD5_RED_ to assess the effect of linear HD5 on LPS-induced inflammation. Compared with HD5_OX_, ^Acm^HD5_RED_ sharply decreased the release of the pro-inflammatory cytokine TNF-α from mouse macrophage RAW264.7 cells (Fig. 7B). Additionally, the accumulation of TNF-α in the cytoplasm of cells treated with ^Acm^HD5_RED_ was markedly lower than that observed in the cells treated with HD5_OX_ (Fig. 7C). Quantitative real-time polymerase chain reaction (RT-PCR) assay further revealed that ^Acm^HD5_RED_ could suppress the production of TNF-α at the transcriptional level (Fig. 7D). Collectively, these findings suggest that HD5_RED_ may act as an immunoregulator, possiblely by blocking the initiation of TLR4 signalling via inhibition of the interaction between LPS and LBP.

Notably, the role of LBP in LPS-TLR4 signalling is conflictive, as low concentrations of LBP are able to mediate the binding of LPS to membrane CD14, but high concentrations of LBP may mediate the transference of LPS to lipoproteins, which attenuates the stimulation of LPS to TLR4 signalling^49^,^50^. Because the expression of LBP in intestine is increased upon the stimulation of inflammatory mediators^51^, it was previously proposed that the local production of low concentrations of LBP by epithelial cells was to enhance the sensitivity of the immune system to LPS, thus resulting in high local concentrations of LBP to neutralize LPS^52^. Our findings of the immuoregulatory effect of HD5_RED_ provide an interesting supplement to the relationship between LBP and LPS-stimulated inflammation. As HD5_RED_ is a component of the innate immunity, when LPS is released from bacteria, HD5_RED_ binds LPS and blocks the interaction between LPS and low concentrations of LBP at the early stage, which suppresses the downstream cascade reaction, including the release of inflammatory mediators. However, when the release of LPS surpasses the neutralization of HD5_RED_, the increasing content of inflammatory mediators induces the expression of LBP from intestinal epithelia, which then transfers excrescent LPS to lipoproteins and lowers the inflammatory responses. Therefore, we presume that HD5_RED_ and high concentrations of LBP are both beneficial to the intestinal homeostasis.

Normally, the *Bacteroides* and *Parabacteroides* genera, two Gram-negative rods, constitute approximately 30% of the bacterial load in the gut^53^, representing a heavy burden in terms of LPS stimuli for host cells. In healthy individuals, because LPS can induce the expression of Trx^54^, and because Trx has the ability to catalyze the reduction of HD5_OX_, there might be a balance between HD5_OX_ and HD5_RED_, which plays an undetected role in controlling LPS-induced inflammation. In patients with IBD, radiation-induced intestine injury, or other enteric diseases, the content of HD5 and the balance between HD5_RED_ and HD5_OX_ in the enteric canal might be altered. The pathological significance of the balance needs further investigation in the future.

## Conclusions

**Figure 8:**
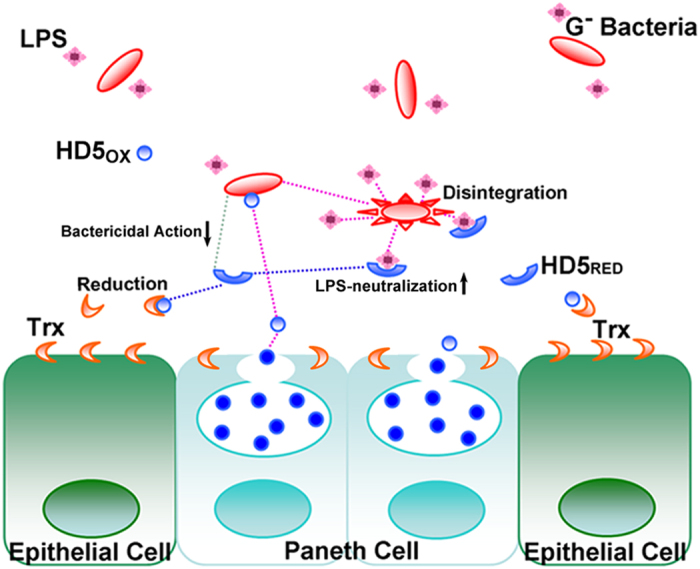
Schematic drawing illustrating the generation of HD5_RED_ and the function of HD5_RED_ against Gram-negative bacteria in the intestine.

As shown in Fig. 8, Paneth cell-secreted HD5_OX_, which contains three specific disulfide bonds, can strongly target and destroy microbes and increase LPS release, which, if left unchecked, would induce a severe inflammatory response. Consequently, partial HD5_OX_ peptides are reduced by Trx to produce HD5_RED_. HD5_RED_ is less effective in killing bacteria but more potent in binding LPS and blocking the interaction between LPS and low concentrations of LBP, thus suppressing the activation of TLR4-signalling and decreasing the release of pro-inflammatory cytokines that benefit the host in a manner that is complementary to HD5_OX_.

## Materials and Methods

### Peptide preparation

Peptides were all prepared by Chinese Peptide Company (FDA 3004518843). HD5_OX_ and ^Acm^HD5_RED_ were synthesized using a machine-assisted solid-phase chemical method^55^. HD5_OX_ has been used in our previous study^10^. HD5_RED_ was prepared by incubating 1 mg/mL of HD5_OX_ with 40 mM dithiothreitol (DTT) at 37 °C for 2 h. Peptides were all purified using an Agilent 1260 HPLC system equipped with a Phenomenex/Luna C18 column (5 μm, 4.6 × 150 mm) and dried by vacuum-centrifuge. HD5_RED_ was stored under argon gas at −20 °C. After receiving the peptides, we determined their purities and molecule masses by reverse-phase high performance liquid chromatography (RP-HPLC) and matrix-assisted laser desorption/ionization-time of flight mass spectrometry (MALDI-TOF), respectively, which were shown in Supplementary Fig. 6. Peptides were prepared in sterile water, with a storage concentration of 1 mg/mL. To protect HD5_RED_ from oxidation, 20 mM DTT was added into the solution.

### Immunofluorescence microscopy

Immunofluorescence microscopy was carried out with a Zeiss LSM780 laser scanning confocal microscope. The terminal ileum samples were obtained from a healthy donor underwent enteroscopy. The experiment was approved by the Third Military Medical University Institutional Review Board. A rabbit polyclonal anti-HD5 antibody was prepared according to a published study^13^. HD5_RED_ (1 mg) conjugated to the keyhole limpet haemocyanin (KLH) was used as the immunogen. The antisera collected at the 7^th^ week were initially purified by GE HiTrap Protein G and HD5_RED_-containing HP columns, where the GE NHS-activated Sepharose 4 Fast Flow was used. To eliminate the cross reaction of antibodies recognizing KLH, the eluant was further purified by a KLH-containing HP column, where the non-specific antibodies were covalently bound. Specificity of the remaining antibody was verified by an enzyme-linked immunosorbent assay (ELISA) (Supplementary Table 2). Citrate buffer (10 mM, pH 6.0) was used for heat-induced antigen retrieval. The frozen sections were incubated with a normal rabbit IgG (Boster BA1041, 1:200), the rabbit polyclonal anti-HD5 antibody (1:200), and a rabbit polyclonal anti-thioredoxin (Trx) antibody (Abcam ab86255, 1:200), respectively. The goat anti-rabbit Alexa Fluor 488 was employed to determine the location of HD5 and Trx.

To detect TNF-α in the cytoplasm of murine macrophage RAW264.7 cells, the cells (1 × 10^5^/well) were cultured on glass cover slips in DMEM supplemented with 10% FCS. After adherence, the cells were then washed and incubated with LPS (Sigma L2880, 5 ng/mL) in the absence or presence of peptides (50 μg/mL, pre-incubated at 37 °C for 30 min). A primary antibody (Abcam ab1793, 1:100) and a goat anti-mouse secondary antibody (Invitrogen, Alexa Fluor 555) were subsequently used to stain TNF-α. Nuclei were stained using DAPI.

### HD5_OX_ reduction

A mixture of 30 μM HD5_OX_, 15 μM human thioredoxin (Trx, Prospec PRO569), 500 nM rat liver thioredoxin reductase (TrxR, Sigma T9698), 1 mM NADPH (Sigma N5130), and 2 mM EDTA was incubated in 100 mM sodium phosphate buffer (pH 7.0) at room temperature for 1 h. The reaction was quenched by adding 6% trifluoroacetic acid (TFA). The solution was then centrifuged (10,000 g × 10 min) at 4 °C and analyzed with an Agilent 6410 liquid chromatography-mass spectrometry (LC-MS) instrument using a linear gradient of 5–45% acetonitrile that contained 0.1% TFA.

### Acetic acid urea-polyacrylamide gel electrophoresis (AU-PAGE) immunoblotting

The luminal fluid (LF) samples were obtained from the terminal ileum of four healthy donors underwent enteroscopy. For each donor, approximately 2 mL of the LF was collected using a sterile fine catheter. Once removed, it was alkylated by incubating with 50 mM iodoacetamide (IAM) at room temperature for 1 h, and the samples were subsequently processed as previously described^6^. Briefly, it was initially diluted to a final concentration of 20% (v/v) by acetic acid. The protease inhibitor cocktail (1:100, v/v) was added, and the precipitates were removed by centrifugation (12,000 g × 15 min) at 4 °C. The supernatant was then diluted 1:19 (v/v) with 5 mM ammonium acetate (pH 6.0) and purified by the GE ÄKTApurifier equipped with a HiTrap CM FF column at a running speed of 0.5 mL/min. Peptides were eluted by 5 mM ammonium acetate containing different concentrations of NaCl, which was further subjected to desalination by a HiPrep 26/10 Desaltiing column.

The eluants were lyophilized and dissolved with 200 μL of 5% acetic acid, which were named as LF1, LF2, LF3, and LF4, respectively. A total of 20 μL of each sample was analyzed on a 15% AU-PAGE gel. HD5_OX_ and ^IAM^HD5_RED_ (500 ng/lane) were employed as the positive controls. ^IAM^HD5_RED_ was prepared by incubating 500 ng HD5_RED_ with 1 mM IAM at room temperature for 2 h. The electrophoresis was carried out in 5% acetic acid, and methyl green was used as the tracking dye. Proteins were transferred to a Millipore PVDF membrane (0.22 μm) with a Bio-Rad semi-dry transfer cell at 1.5 mA/cm^2^ for 40 min. The membrane was subsequently blocked in 5% skim milk and incubated with the rabbit polyclonal anti-HD5 antibody (1:100) at 4 °C over night. Protein bands were displayed with the Pierce ECL Plus regent (Thermo 32132).

### LF MALDI-TOF

The α-cyano-4-hydroxy cinnamic acid (CHCA) was prepared in a solution containing 50% acetonitrile and 0.2% TFA, with a final concentration of 10 mg/mL. Aliquot of 1 μL of LF2 was mixed with 1 μL CHCA and loaded on the sample plate. HD5_OX_ and ^IAM^HD5_RED_ were employed as the positive controls. Co-crystallization was actualized at room temperature. The mass spectrometric analysis was performed in the linear mode on a time-of-flight mass spectrometer (MALDI 7090, Shimadzu). The molecular masses ranged from 1,000 to 4,500 Da were obtained and processed by MALDI Solutions Data Acquisition software.

### Antibacterial assay

A virtual colony count protocol^7^ for *Salmonella enterica* serovar Typhimurium (*S.* Typhimurium, ATCC 14028), *Escherichia coli* (*E. coli*, ATCC 25922), *E. coli* ML35 (ATCC 43827), and *Enterococcus faecalis* (*E. faecalis*, ATCC 29212) was used. Bacteria were cultured in Tryptic Soytone Broth (TSB) until they reached the mid-logarithmic-phase and were then diluted to 1 × 10^6^ CFU/mL with 10 mM sodium phosphate buffer (pH 7.4) containing 1% TSB. The peptides were prepared by two-fold serial dilution from 250 to 7.8 μg/mL in sterile water. To determine the influence of 4 μg/mL of LPS on the antibacterial activities of peptides against *E. coli*, the concentration of peptides was fixed at 125 μg/mL. Mixtures of 90 μL of bacteria and 10 μL of the peptides (or sterile water in the absence and presence of DTT) were co-incubated at 37 °C for 1 h, and the reaction was then quenched by adding 100 μL of doubly concentrated Mueller-Hinton broth. Bacterial growth was monitored using a Molecular Devices M2e microplate reader. A Visual Basic program was developed to facilitate the analysis. This assay was conducted in duplicate and repeated three times.

### Zeta potential determination

*E. coli* ML35 (ATCC 43827) cells grown to the mid-logarithmic phase were centrifuged, resuspended, and diluted to the absorbance (600 nm) of 0.2 in 10 mM sodium phosphate buffer (pH 7.4) containing 1% TSB. A total of 900 μL of the cells and 100 μL of the peptides (or sterile water in the absence and presence of 1 mM DTT) were incubated and loaded into a disposable zeta cell with gold electrode. Samples were equilibrated at 25 °C for 2 min. Zeta potential was determined using the Zetasizer Nano ZS (Malvern Instruments, UK). A total of 6 measurements of 100 runs were carried out for each sample. This assay was repeated twice and the data were analyzed for statistical significance using a Student−Newman−Keuls multiple comparisons test.

### Biolayer interferometry (BLI)

The recruitment of peptides to lipid A (Sigma L5399) was determined using Forte Bio’s “Octet Red 96” BLI (Pall Life Sciences, US). The amine-reactive second-generation (AR2G) biosensor was activated using 20 mM 1-ethyl-3-[3-dimethylaminopropy] carbodiimide hydrochloride (EDC, Sigma E1769) and 10 mM sulfo-N-hydroxysulfosuccinimide (s-NHS, Sigma 56485). Lipid A was immobilized on biosensors at the concentration of 20 μg/mL until the binding curves reached the plateau. Ethanolamine (1 M, pH 8.5) was used to quench the immobilization. Peptides were prepared in the running buffer (5 mM sodium phosphate buffer, pH 7.4), with concentrations of 200, 400, 600, 800, 1000, and 1200 nM, respectively. Association and disassociation, 5 min for each, were carried out at a shaking speed of 600 rpm. Biosensors were regenerated by 0.1 M Glycine-HCl buffer (pH 2.0). The specificity of lipid A interacting with peptides was demonstrated by an experiment using 800 nM peptides (Supplementary Fig. 7). The binding thicknesses were processed using Fortebio Data Analysis 7.0 software, in which the methods for y-axis alignment, inter-step correction, and Savitzky-Golay filtering were specified. The equilibrium dissociation constant (K_D_), calculated as the ratio of the dissociation rate constant (K_off_) to the association rate constant (K_on_), was generated by a 1:1 fitting model. This experiment was repeated three times.

### Outer membrane penetration

A 1-N-phenylnaphthylamine (NPN) uptake assay was performed to probe the lipid exposure of the *E. coli* ML35 (ATCC 43827) outer membrane. Bacteria grown to the mid-logarithmic phase were centrifuged and resuspended in 10 mM sodium phosphate buffer (pH 7.4). Mixtures of 190 μL cells (1 × 10^8^ CFU/mL) and 10 μL peptides (or sterile water in the absence and presence of 0.13 mM DTT) were co-incubated at 37 °C for 1 h. Then, an aliquot of 10 μL of NPN (Sigma 104043) was added, resulting in a final concentration of 10 μM. After excitation at 350 nm, the fluorescent intensity was measured in the range from 380–450 nm at intervals of 2 nm using a Tecan Infinite M1000 Pro microplate reader. This assay was conducted in duplicate and repeated three times.

### Inner membrane permeabilization

A total of 80 μL of *E. coli* ML35 (1 × 10^7^ CFU/mL) grown to the mid-logarithmic phase, 10 μL 2-nitrophenyl β-D-galactopyranoside (ONPG, Sigma N1127, 25 mM), and 10 μL peptides (or sterile water in the absence and presence of 0.25 mM DTT) were co-incubated at 37 °C for 2 h with 50 s of shaking every 5 min. The production of 2-nitrophenol (ONP), a marker of inner membrane penetration^10^, was determined by measuring the absorbance at 405 nm. This experiment was conducted in duplicate and repeated three times.

### Membrane depolarization

*E. coli* ML35 cells grown to the mid-logarithmic phase were diluted with 10 mM sodium phosphate buffer (pH 7.4) to 5 × 10^6^ CFU/mL. A total of 180 μL of the cells were exposed to 20 μL of peptides (or sterile water in the absence and presence of 0.25 mM DTT) at 37 °C for 1 h, and the mixture was then incubated with 1 μg/mL bis-(1,3-dibutylbarbituric acid) trimethine oxonol [DiBAC4(3), Dojindo D545] for 10 min. DiBAC4(3) is a potential-sensitive anionic dye that passes into cells with depolarized membranes according to the Nernst equation, resulting in increased fluorescence that can be detected using flow cytometry^13^,^56^. The bacteria were subsequently centrifuged (4,500 g, 10 min) and resuspended in 300 μL cold PBS for two times. The percentage of depolarized cells was measured using a BD FACSVerse flow cytometer, and 50,000 events per sample were obtained. By analyzing the FSC-H/FSC-W and SSC-H/SSC-W scatterplots, the widened splashes of cells treated with peptides relative to cells treated with sterile water were discarded. The data were processed using FlowJo software (version 7.6.1) and analyzed for statistical significance using a Student−Newman−Keuls multiple comparisons test.

### Circular dichroism (CD) spectroscopy

The conformational properties of the peptides were analyzed using an Applied Photophysics Chirascan instrument at 27 °C. A cell with a 1-mm path length was utilized. The CD spectra were obtained from 190–280 nm at 1 nm intervals. The time-per-point was 0.5 s, and the scanning time was approximately 73 s. Peptides (100 μg/mL, 250 μL) prepared in sterile water were examined in the presence of 20 mM sodium dodecyl sulfate (SDS) and 100 μg/mL LPS. Before scanning, peptides were incubated with SDS and LPS at room temperature for 1 h. The data obtained from three independent scans were averaged and smoothed by Pro-Data Chirascan software (version 4.1).

### Isothermal titration calorimetry (ITC)

Interactions between peptides and LPS were monitored using a MicroCal ITC200 system (Malvern, UK) at 25 °C. The concentration of the peptides in the syringe was 1 mg/mL (40 μL). LPS was prepared in sterile water at a concentration of 10 μg/mL (300 μL per cell). Twenty injections (0.2 μL for the first injection and 2 μL for the remaining injections) were applied at 120 s intervals and at a stirring rate of 1,000 rpm. The data were processed by subtracting the baseline heat release from titrations of sterile water into LPS and from 20 mM DTT into LPS. The experiment was repeated three times.

### LPS-binding protein (LBP) block assay

The effects of peptides on the interaction between LPS and low concentrations of LBP were determined as previously described^20^. Briefly, the plates of a LBP ELISA kit (Boster EK1274) were blocked by the incubation of 1% BSA prepared in 10 mM sodium phosphate buffer (pH 7.4) at 37 °C for 1 h, which were subsequently washed with 0.1% Tween-20. Aliquots of 100 μL of mouse LBP (Sigma SRP6034, 60 ng/mL) were added and incubated at 37 °C for 1.5 h. Peptides were prepared in sterile water, with concentrations of 12.5, 25, and 50 μg/mL, respectively. Biotinylated LPS (50 ng/mL) was added in the absence or presence of peptides (pre-incubated at 37 °C for 30 min). Polymyxin B (200 ng/mL) was used as a positive control. The binding of LPS to LBP was determined using a chromogenic reaction between HRP-conjugated streptavidin (Boster BA1088) and TMB (Sigma 860336). Data were processed by subtracting the absorbance in the absence of LPS. Statistical significance was determined by the Student−Newman−Keuls multiple comparisons test. The experiment was conducted in duplicate and repeated three times.

### TNF-α release detection

The murine macrophage cell line RAW264.7 was obtained from the cell bank of Chinese academy of sciences (CAS, Shanghai). Cells were grown in DMEM containing 10% FCS, 2 mM L-glutamine, and 1 mM sodium pyruvate, seeded into the wells of a 24-well plate at a density of 5 × 10^5^ cells/well and cultured at 37 °C overnight. The cells were then washed and incubated with LPS (5 ng/mL) for 9 h in the absence or presence of peptides (50 μg/mL, pre-incubated at 37 °C for 30 min). The TNF-α content in the supernatant was determined by ELISA (Boster EK0527). Statistical comparisons of the data were performed using a Student−Newman−Keuls multiple comparisons test. This assay was performed in duplicate and repeated three times.

### Quantitative real-time polymerase chain reaction (RT-PCR)

The RNA of RAW264.7 cells was extracted using the TaKaRa (Dalian, CHN) RNAiso Plus reagent. A total of 900 ng of RNA was reverse-transcribed using a TaKaRa PrimeScript^TM^ RT-PCR kit (DRR014A). The following forward (F) and reverse (R) primers were used: TNF-α: F, 5′-GGAACTGGCAGAAGAGGC-3′; R, 5′-CACTTGGTGGTTGCTACG-3′; and β-actin, F, 5′-GAGACCTTCAACACCCCAGC-3′; R, 5′-ATGTCACGCACGATTTCCC-3′. Data were obtained using Bio-Rad iQ5 standard edition optical system software (version 2.1) and analyzed for statistical significance using a Student−Newman−Keuls multiple comparisons test. The experiment was conducted in duplicate and repeated three times.

## Additional Information

**How to cite this article**: Wang, C. *et al.* Reduction Impairs the Antibacterial Activity but Benefits the LPS Neutralization Ability of Human Enteric Defensin 5. *Sci. Rep.* **6**, 22875; doi: 10.1038/srep22875 (2016).

## Supplementary Material

Supplementary Information

## Figures and Tables

**Table 1:** Lethal Doses (LDs, μg/mL) that Kill 50% and 90% of Bacteria.

	HD5_OX_		HD5_RED_	
	LD50	LD90	LD50	LD90
ATCC 14028 *S.* Typhimurium	4.6 ± 0.5	9.6 ± 0.7	7.9 ± 1.1	19.5 ± 1.6
ATCC 25922 *E. coli*	3.2 ± 0.7	7.6 ± 1.1	6.5 ± 0.8	12.9 ± 1.3
ATCC 43827 *E. coli* ML35	4.7 ± 0.8	11.6 ± 1.7	9.9 ± 0.6	22.5 ± 2.3
