# Supplementary material for: Reduction Impairs the Antibacterial Activity but Benefits the LPS Neutralization Ability of Human Enteric Defensin 5
Source: Sci Rep. 2016 Mar 10;6:22875. doi: 10.1038/srep22875 (PMC4785407; doi:10.1038/srep22875)
Supplement: Supplementary Information [file srep22875-s1.doc]

**Supplementary Information**

**Reduction Impairs the Antibacterial Activity but Benefits the LPS Neutralization**

**Ability of Human Enteric Defensin 5**

Cheng Wang^1#^, Mingqiang Shen^1#^, Naixin Zhang^1^, Song Wang^1^, Yang Xu^1^, Shilei Chen^1^, Fang Chen^1^, Ke Yang^1,2^, Ting He^2^, Aiping Wang^1^, Yongping Su^1^, Tianmin Cheng^1^,

Jinghong Zhao^2^, Junping Wang^1*^

1. State Key Laboratory of Trauma, Burns and Combined Injury, Institute of Combined Injury of PLA, Chongqing Engineering Research Center for Nanomedicine, College of Preventive Medicine, Third Military Medical University, Chongqing, 400038, China

2. Department of Nephrology, Xinqiao Hospital, Third Military Medical University, Chongqing, 400037, China

Contents:

Table 1: The equilibrium dissociation constant values of peptides binding to lipid A (**S2**)

Table 2: Enzyme-linked immunosorbent assaya (ELISA) demonstrating the specificity of the rabbit polyclonal anti-HD5 antibody (**S3**)

Figure 1: The full-length blots illustrating the existence of ^IAM^HD5_RED_ in IAM-alkylated luminal fluid (LF) (**S4**)

Figure 2: Co-loading ^IAM^HD5_RED_ and LF resulted in a single but wider band in AU-PAGE immunoblotting (**S5**)

Figure 3: HD5_OX_ is more efficient in killing *Enterococcus faecalis* than HD5_RED_ (**S6**)

Figure 4: The relationship between the enthalpy value (△H) of the self-association of HD5_OX_ and the molar ratio (**S7**)

Figure 5: The efficiencies of peptides in blocking the interaction between LPS and LBP (**S8**)

Figure 6: Determination of the purities and molecular masses of peptides (**S9**)

Figure 7: The specific interaction between peptides and lipid A loaded on biosensors (**S10**)

**S1**

**Table 1. The equilibrium dissociation constant values of peptides binding to lipid A*^a^***

| **Peptides** | **Repeat** | **Kon*^b^* (1/Ms)** | **Koff *^b^* (1/s)** | **KD*^b^* (nM)** | **R^2^ *^b^*** | **KD** **(statistics, nM )** |
| --- | --- | --- | --- | --- | --- | --- |
| **HD5_OX_** | 1th | 6.32×10^3^ | 3.24×10^-4^ | 51.3 | 0.9769 | 75 ± 24 |
|  | 2th | 5.41×10^3^ | 5.36×10^-4^ | 99.1 | 0.9832 |  |
|  | 3th | 6.83×10^3^ | 5.09×10^-4^ | 74.5 | 0.9942 |  |
| **HD5_RED_** | 1th | 2.14×10^3^ | 3.89×10^-4^ | 181.8 | 0.9704 | 140 ± 30 |
|  | 2th | 2.57×10^3^ | 3.37×10^-4^ | 131.3 | 0.9902 |  |
|  | 3th | 2.25×10^3^ | 2.64×10^-4^ | 117.3 | 0.9835 |  |

*^a^* The binding kinetics between peptides and bacterial lipid A were determined with a biolayer interferometry (BLI). Lipid A was immobilized on AR2G biosensors at the concentration of 20 μg/mL until the binding curves reached the plateau. Peptides were prepared in the running buffer (5 mM sodium phosphate buffer, pH 7.4), with concentrations of 200, 400, 600, 800, 1000, and 1200 nM, respectively. Association and disassociation, 5 min for each, were carried out at a shaking speed of 600 rpm. The bindings were processed using Fortebio Data Analysis 7.0 software, which specifies the methods for y-axis alignment, inter-step correction, reference subtraction, and Savitzky-Golay filtering. The experiment was repeated three times.

*^b^* K_on_, the association rate constant; K_off_, the dissociation rate constant; K_D_, the equilibrium dissociation constant; R^2^, the fitting correlation coefficient. These parameters were generated by a 1:1 fitting model. K_D_ is calculated as the ratio of K_off_ to K_on_.

**S2**

**Table 2. Enzyme-linked immunosorbent assay*^a^* (ELISA) demonstrating the specificity of the rabbit polyclonal anti-HD5 antibody**

| **Immunogen** | **N** | **P** | | | | | |
| --- | --- | --- | --- | --- | --- | --- | --- |
|  |  | **1 : 5,000 *^b^*** | **1 : 25,000** | **1 : 125,000** | **1 : 625,000** | **1 : 3,125,000** | **1:15,625,000** |
| **KLH** | 0.056 ± 0.002 | **0.091 ± 0.002** | 0.066 ± 0.003 | 0.057 ± 0.002 | 0.051 ± 0.001 | 0.048 ± 0.002 | 0.05 ± 0.004 |
| **KLH-HD5_RED_** | 0.054 ± 0.001 | 1.433 ± 0.122 | 1.263 ± 0.087 | 0.84 ± 0.026 | 0.426 ± 0.039 | **0.201 ± 0.006** | 0.074 ± 0.003 |
| **KLH-^IAM^HD5**_RED_ | 0.053 ± 0.001 | 1.357 ± 0.095 | 1.162 ± 0.073 | 0.774 ± 0.058 | 0.383 ± 0.051 | **0.176 ± 0.007** | 0.061 ± 0.002 |
| **KLH-HD5_OX_** | 0.054 ± 0.001 | 0.981 ± 0.078 | 0.706 ± 0.052 | 0.423 ± 0.048 | **0.19 ± 0.006** | 0.086 ± 0.005 | 0.051 ± 0.001 |
| **KLH-HD6_OX_** | 0.055 ± 0.001 | **0.106 ± 0.004** | 0.084 ± 0.003 | 0.073 ± 0.003 | 0.057 ± 0.002 | 0.045 ± 0.001 | 0.049 ± 0.001 |

*^a^* Approximately 5 μg/mL of keyhole limpet haemocyanin (KLH), HD5_RED_ conjugated to KLH, ^IAM^HD5_RED_ conjugated to KLH, HD5_OX_ conjugated to KLH, and HD6_OX_ (DEFS-008C, Chinese Peptide Company) conjugated to KLH were coated on a Nunc ELISA plate and incubated at 4°C overnight. The plate was blocked by the incubation of 2% BSA prepared in PBS at 37°C for 2 h, which was subsequently washed with PBST (0.1% Tween-20). Antibodies (1 mg/mL) were diluted (1:5,000, 1:25,000, 1:125,000, 1:625,000, 1:3,125,000, 1:15,625,000, respectively) in PBST. Aliquots of 100 μL of the diluted antibodies were added to the wells and incubated at 37°C for 1 h. The plate was then washed with PBST and incubated with an Abcam goat anti-rabbit antibody (HRP-conjugated, ab6721, 1:3000) at 37°C for 30 min. Absorbance at 450 nm was determined after the incubation of TMB (Sigma 860336, 37°C, 20 min) and sulfuric acid. Shown is the result of three independent experiments.0000000000000000000000000000000000000000000000000000000000000000000000000000000000000000000000000000000000000000000000000000000

*^b^* The antibody titer is determined according to P/N > 2.1. P, absorbances of the groups treated with antibodies (positive control); N, absorbances of the groups treated with PBST (negative control). Titers: KLH, > 1:5000; KLH-HD5_RED_, 1:3,125,000; ^IAM^HD5_RED_, 1:3,125,000; KLH-HD5_OX_, 1:625,000; KLH-HD6_OX_, > 1:5000.

**S3**

**
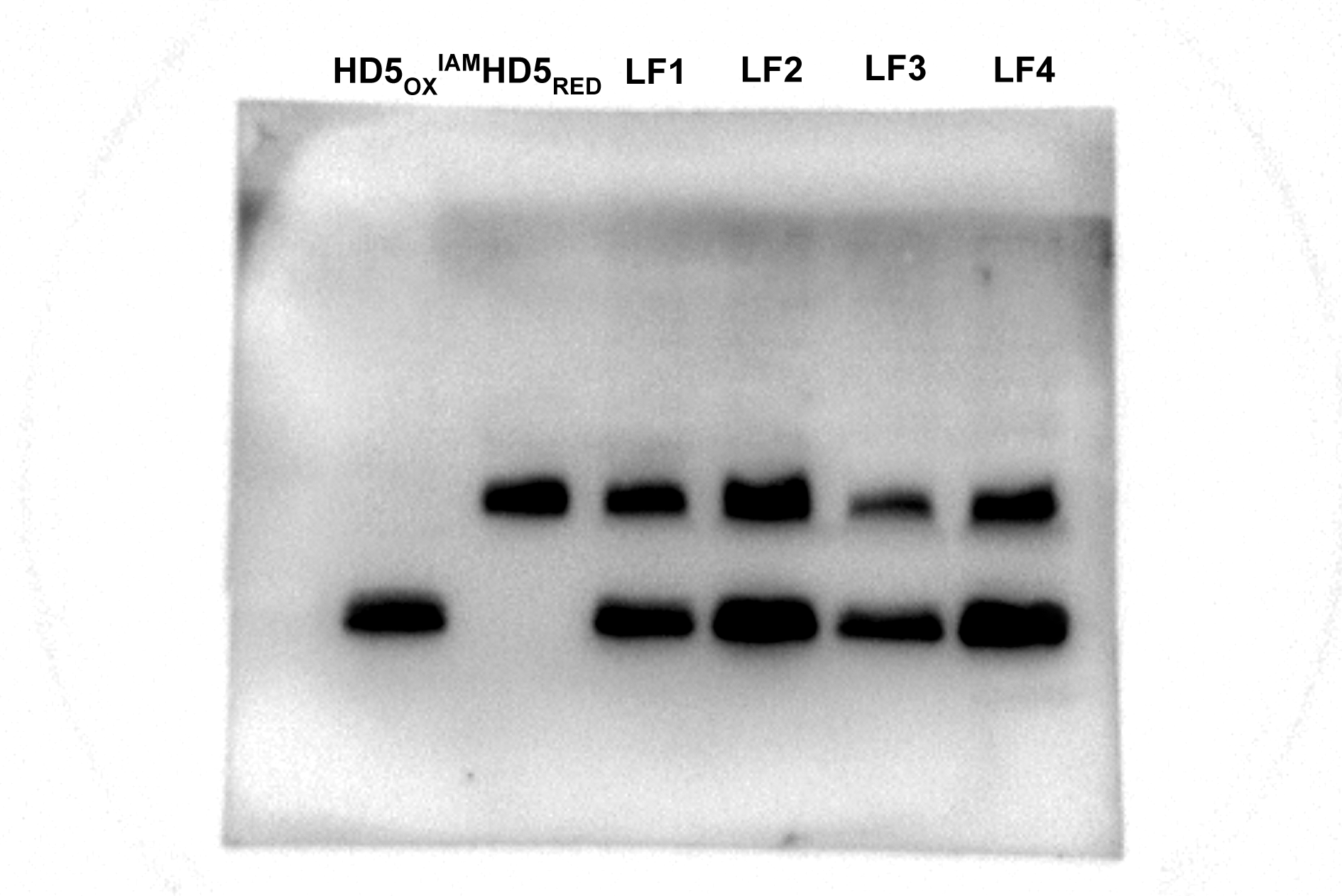
**

**Figure 1. The full-length blots illustrating the existence of ^IAM^HD5_RED_ in IAM-alkylated luminal fluid (LF).** Four LF samples were obtained from the terminal ileum of different donors underwent enteroscope. They were purified, lyophilized, and dissolved with 200 μL of the 5% acetic acid solution, which were named as LF1, LF2, LF3, and LF4, respectively. A total of 20 μL of each sample was resolved by 15% AU-PAGE. HD5_OX_ and ^IAM^HD5_RED_ (500 ng/lane) were employed as the positive controls. Proteins were transferred to a Millipore PVDF membrane (0.22 μm) with a Bio-Rad semi-dry transfer cell at 1.5 mA/cm^2^ for 40 min. The membrane was subsequently blocked in 5% skim milk and incubated with the rabbit polyclonal anti-HD5 antibody (1:100) at 4°C over night. Protein bands were displayed with the Pierce ECL Plus regent (Thermo 32132).

**S4**

**
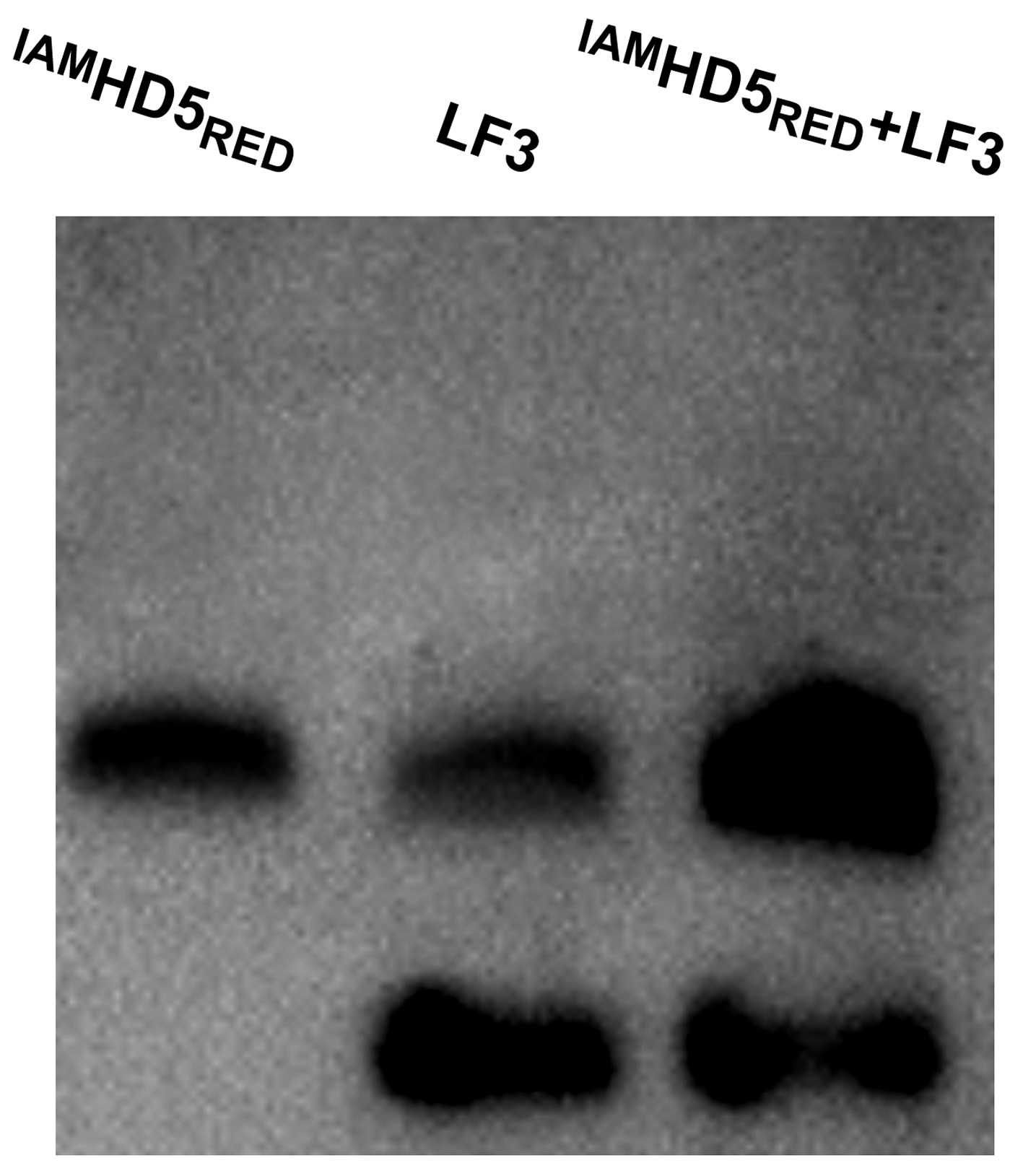
**

**Figure 2. Co-loading ^IAM^HD5_RED_ and LF resulted in a single but wider band in AU-PAGE immunoblotting.** LF was purified, lyophilized, and dissolved with 200 μL of 5% acetic acid solution. Approximately 500ng of ^IAM^HD5_RED_ was employed as a positive control. LF3 (20 μL/lane) was resolved in the presence and absence of ^IAM^HD5_RED_ (500 ng) by 15% AU-PAGE. The experiment was conducted as described in Supplementary Figure 1. The existence of ^IAM^HD5_RED_ in LF was confirmed by that co-loading ^IAM^HD5_RED_ and LF resulted in a single but wider band in the region of ^IAM^HD5_RED_.

**S5**


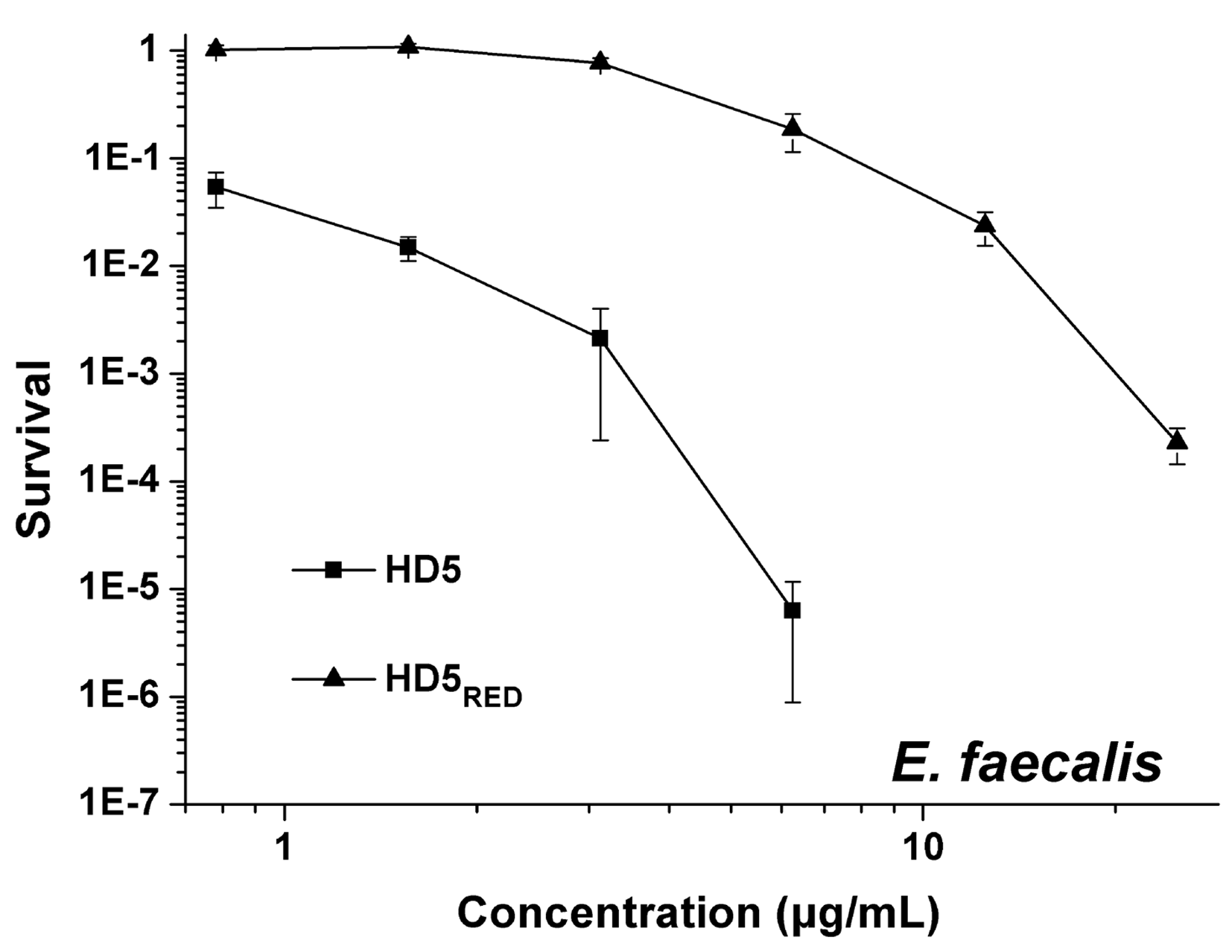


**Figure 3. HD5_OX_ is more efficient in killing *Enterococcus faecalis* than HD5_RED_.** The antibacterial activities of HD5_OX_ and HD5_RED_ against Gram-positive *Enterococcus faecalis* (*E. faecalis*, ATCC 29212) were determined with a virtual colony assay. *E. faecalis* was cultured in TSB until it reached the mid-logarithmic-phase and was then diluted to 1 × 10^6^ CFU/mL with 10 mM sodium phosphate buffer (pH 7.4). HD5_OX_ and HD5_RED_ were prepared by two-fold serial dilution from 250 to 7.8 μg/mL in sterile water. The antibacterial activity is shown as the ratio of the number of surviving colonies after treatment with peptides to the number of surviving colonies after treatment with sterile water (HD5_OX_) or DTT solution (HD5_RED_, 0.016, 0.032, 0.063, 0.13, 0.25, and 0.5 mM DTT, respectively). The results are presented as the mean ± SD. Zero survival points are not plotted.

**S6**


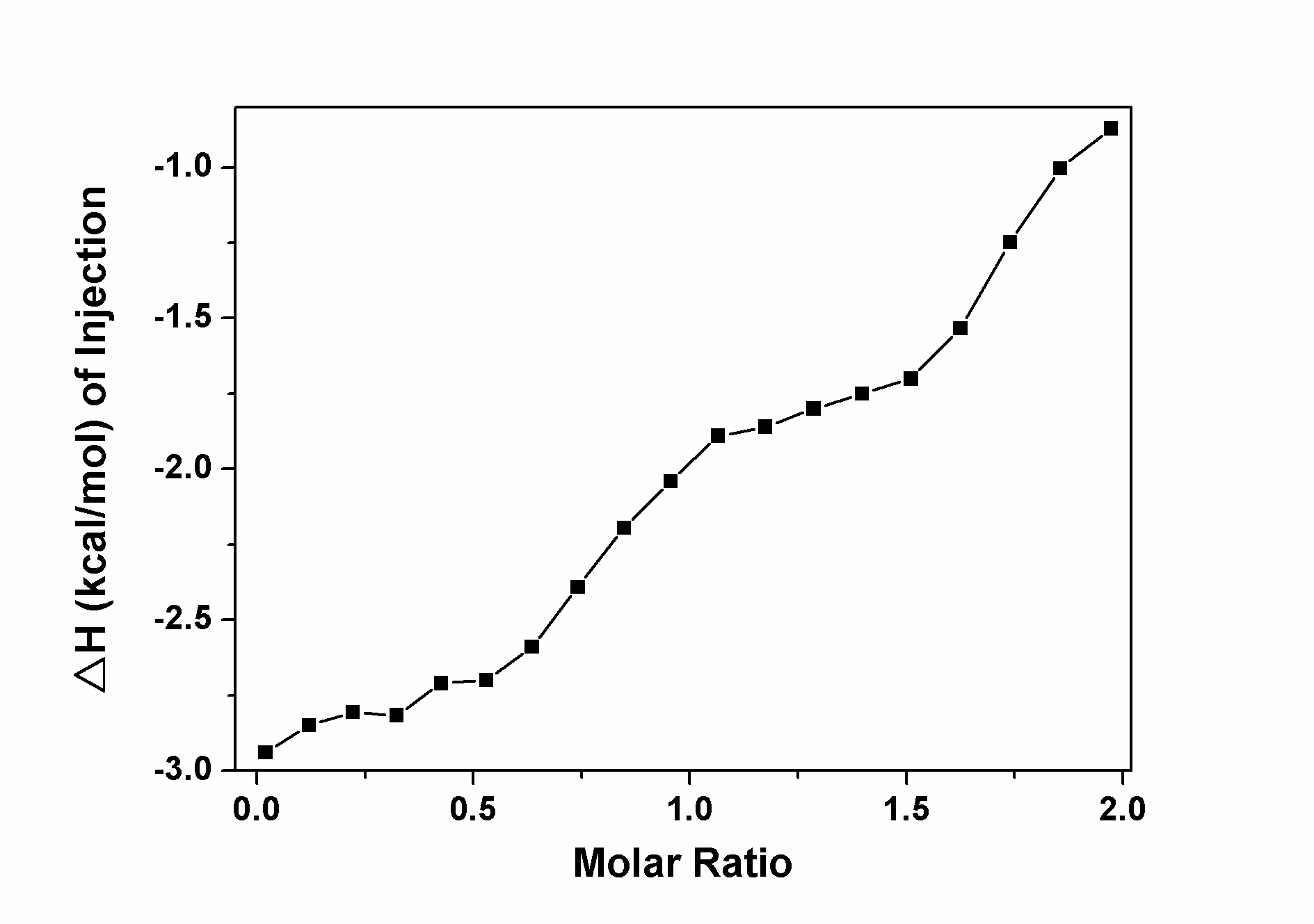


**Figure 4. The relationship between the enthalpy value (△H) of the self-association of HD5_OX_ and the molar ratio.** HD5_OX_ peptide dimerizes in a dose-dependent manner. HD5_OX_ in the syringe were prepared in sterile water with a concentration of 1mg/mL, which was 10-fold higher than the corresponding concentration in the cell. A total of 20 injections were used; the volumes of the solution were 0.2 μL for the first injection and 2 μL for the remaining 19 injections. The separation between injections was 120 s. Shown is a representative relationship between the △H and the molar ratio of HD5_OX_. Along with the saturation of the binding interaction, the △H progressively decreased.

**S7**


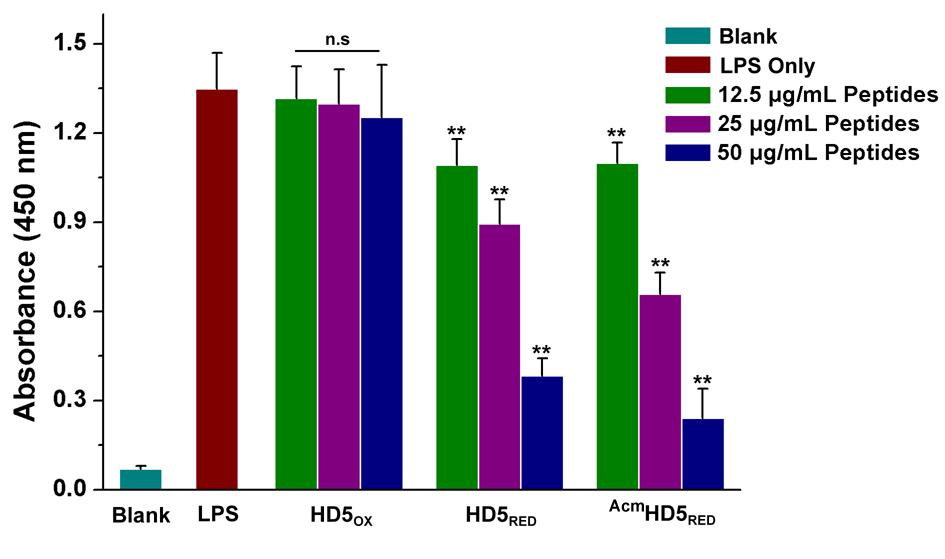


**Figure 5. The efficiencies of peptides in blocking the interaction between LPS and LBP.** A LBP ELISA plate was blocked by 1% BSA and subsequently washed with 0.1% Tween-20. Aliquots of 100 μL of the mouse LBP (60 ng/mL) was added and incubated at 37°C for 1.5 h. Peptides were prepared in sterile water, with concentrations of 12.5, 25, and 50 μg/mL, respectively. Biotinylated LPS (50 ng/mL) was then added in the absence or presence of peptides (pre-incubated at 37°C for 30 min). Data were processed by subtracting the absorbance in the absence of biotinylated LPS. Statistical significance was determined by the Student−Newman−Keuls multiple comparisons test. n.s, not significant; **, p < 0.01, relative to the value of the group solely treated with LPS.

**S8**

**
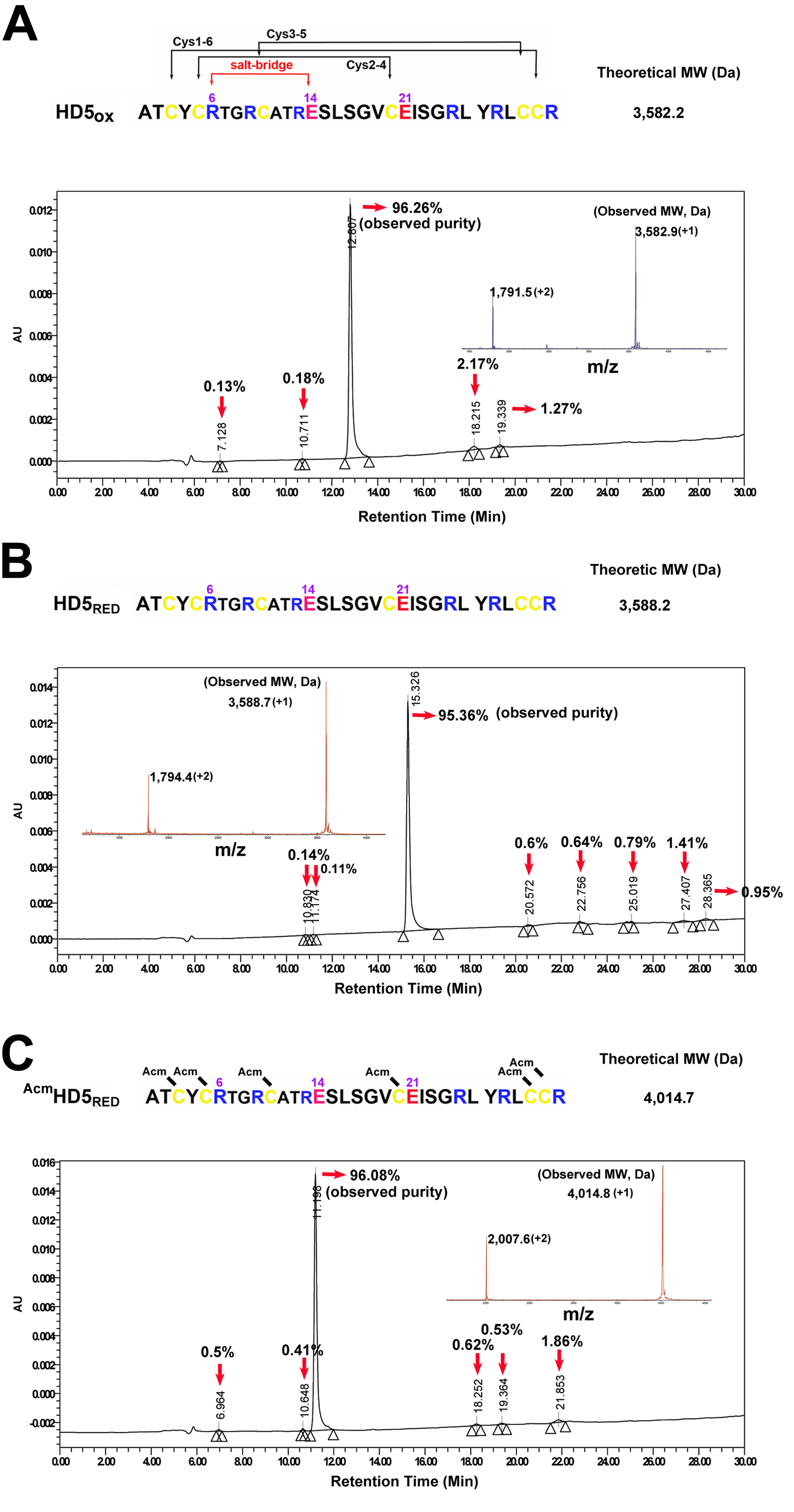
**

**Figure 6. Determination of the purities and molecular masses of peptides.** After receipt of the lyophilized peptides, we prepared them in sterile water. Approximately 100 μg/mL of the peptides were analyzed by Waters 2695 RP-HPLC equipped with a Kromasil 300-5C18 column (250 × 4.6 mm). The gradient elution was run from solvent A (90% water mixed with 10% acetonitrile containing 0.01% trifluoroacetic acid) to solvent B (90% acetonitrile mixed with 10% water containing 0.01% trifluoroacetic acid) at a flow rate of 0.6 mL/min for 30 min. Purity was determined by the area ratio. The purities were 96.26%, 95.36%, and 96.08% for HD5_OX_ (**A**), HD5_RED_ (**B**), and ^Acm^HD5_RED_ (**C**), respectively. Molecular masses were determined by MALDI-TOF (MALDI 7090, Shimadzu). The observed masses were 3,582.9 Da, 3,588.7 Da, and 4,014.8 Da for HD5_OX_, HD5_RED_, and ^Acm^HD5_RED_, respectively. The theoretical mass was determined by ProtParam (<http://web.expasy.org/protparam/>).

**S9**

**
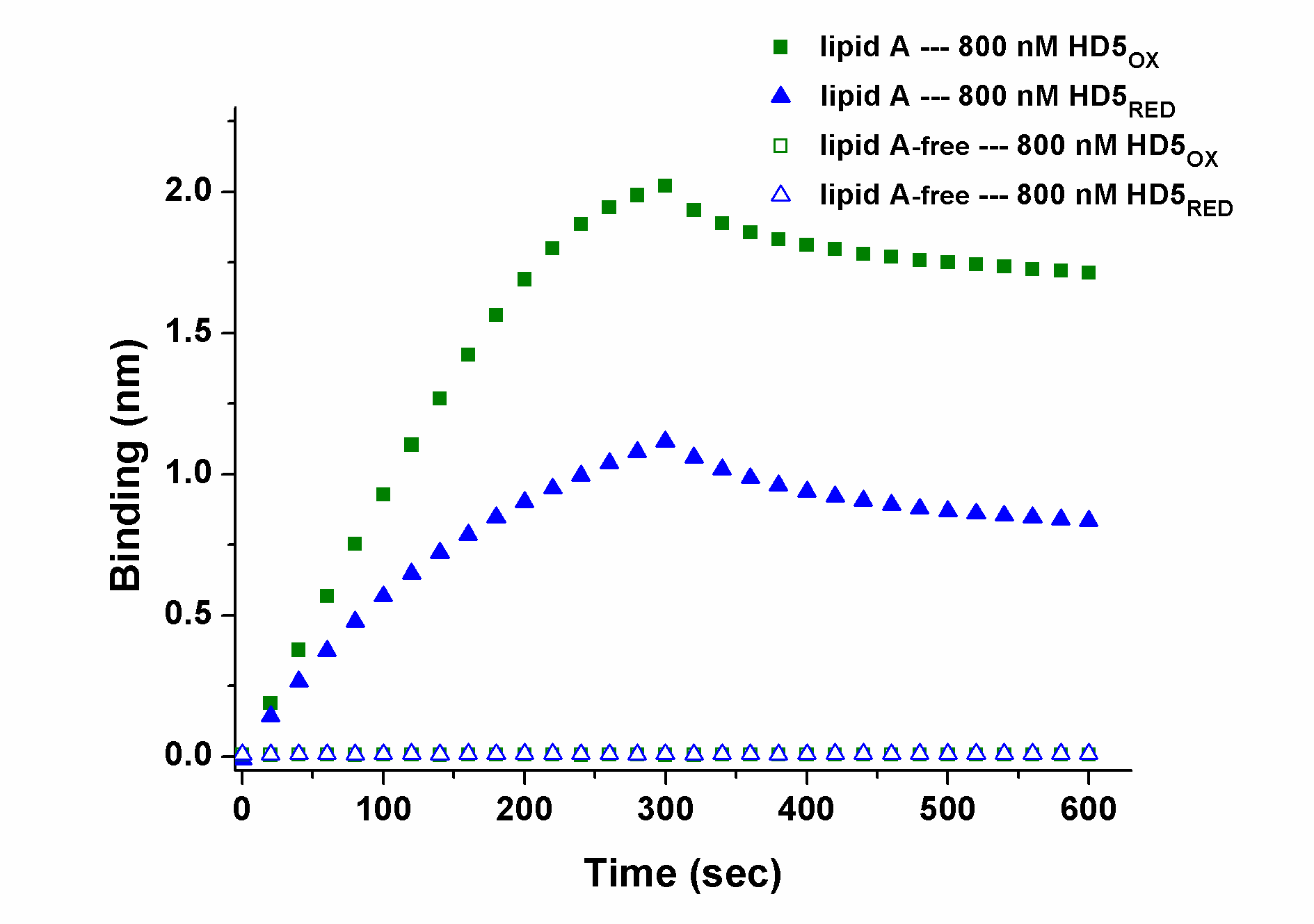
**

**Figure 7. The specific interaction between peptides and lipid A loaded on biosensors.** Four amine-reactive second-generation (AR2G) biosensors were activated in a mixture containing 20 mM 1-ethyl-3-[3-dimethylaminopropy] carbodiimide hydrochloride (EDC) and 10 mM sulfo-N-hydroxysulfosuccinimide (s-NHS) for 5 min. Lipid A was prepared in 10 mM sodium acetate buffer (pH 5), with a concentration of 20 μg/mL. Two of the biosensors interacted with lipid A for 10 min, while the other two solely interacted with 10 mM sodium acetate buffer. These biosensors were subsequently quenched with 1 M ethanolamine (pH 8.5). Peptides were prepared in 5 mM sodium phosphate buffer (pH 7.4), with a final concentration of 800 nM. The interaction between lipid A loaded on biosensors and peptides were detected with a Forte Bio’s “Octet Red 96” biolayer interferometry at a shaking speed of 600 rpm. The specific interaction was confirmed by that only the biosensors interacting with lipid A enabled to recruit peptides.

**S10**
